# Integrating soft skills into perfusion education: a necessity for modern clinical practice

**DOI:** 10.1051/ject/2025047

**Published:** 2026-09-15

**Authors:** Salman Pervaiz Butt, Salman Abdulaziz, Umer Darr, Gopal Bhatnagar

**Affiliations:** 1 Perfusion Services, Heart Vascular and Thoracic Institute, Cleveland Clinic Abu Dhabi PO Box 112412 Abu Dhabi United Arab Emirates; 2 Critical Care Services Administration, King Fahad Medical City 12231 Riyadh Saudi Arabia; 3 Cardiac Surgery, Heart Vascular and Thoracic Institute, Cleveland Clinic Abu Dhabi PO Box 112412 Abu Dhabi United Arab Emirates

**Keywords:** Perfusion education, Non-technical skills, Communication, Conflict management, Simulation, Debriefing, Patient safety

## Abstract

Soft, non-technical competencies are important in perfusion practice but are often inconsistently taught. Key domains include communication, teamwork, leadership, adaptability, emotional intelligence, conflict management, professionalism, and cultural awareness. Suggested methods are interprofessional simulation, structured debriefing with feedback, short courses on communication and conflict resolution, mentored rotations with behavioral checklists, and recognition of progress through competency milestones and continuing professional development. Clear learning objectives and simple, validated assessments can help programs include these elements. Embedding soft-skill expectations in perfusion curricula, appraisal, and certification supports better coordination in the operating room, more consistent crisis management, and stronger collaboration across disciplines and with patients. A steady, stepwise adoption of these methods can improve consistency and patient safety across programs.

To the Editor

The evolving role of the clinical perfusionist in modern healthcare necessitates proficiency not only in technical skills but also in non-technical or “soft” skills. As surgical procedures and team-based care models grow increasingly complex, the ability of perfusionists to communicate effectively, collaborate under pressure, and demonstrate emotional intelligence and professionalism has a direct impact on patient outcomes and team dynamics. Traditionally, these soft skills have been acquired informally through experience, but this passive approach is no longer sufficient in today’s high-stakes clinical environments. Despite the critical importance of these competencies, perfusion education programs remain predominantly focused on technical and physiological training, leaving a gap in structured soft skills development. This paper advocates for the integration of soft skills into perfusion curricula through strategies such as simulation-based training, structured debriefings, formal instruction in communication and conflict resolution, and mentorship during clinical rotations. Additionally, recognition of these skills in certification and continuing education frameworks is essential to standardize and reinforce their value. Proactively integrating soft skills training into perfusion education will better prepare future practitioners to meet the multifaceted demands of modern healthcare, thereby enhancing both patient safety and professional effectiveness.

The role of the clinical perfusionist continues to evolve in parallel with advancements in surgical technique, critical care protocols, and extracorporeal technologies. While technical proficiency and a clear understanding of physiology remain fundamental to the discipline, the relevance of non-technical or “soft” skills has become increasingly apparent. These skills, encompassing communication, teamwork, emotional intelligence, adaptability, conflict resolution, leadership, and professionalism, contribute significantly to the quality and safety of patient care [1].

Historically, the development of soft skills in perfusionists has largely occurred informally. Senior perfusionists often acquire these competencies over many years through repeated exposure to high-pressure clinical environments and ongoing interactions within multidisciplinary teams. This experiential model has undoubtedly shaped many capable and adaptive professionals. However, as clinical demands and team-based care models grow more complex, relying solely on on-the-job learning to develop these critical abilities may no longer be sufficient. In the operating room and intensive care unit, perfusionists routinely participate in time-sensitive decision-making, rapid coordination with surgical and anesthetic teams, and communication under pressure. The effectiveness of these interactions is often determined not by technical knowledge alone, but by interpersonal effectiveness, situational awareness, and the ability to anticipate and respond to the dynamic team’s needs. Inadequate communication or poor conflict resolution in these settings can contribute to workflow inefficiencies, procedural delays, or even compromise patient safety [2].

Soft skills also contribute significantly to other essential domains of perfusion practice, including informed consent discussions (in certain contexts), patient and family interaction during extracorporeal life support (ECLS), crisis management, teaching and mentorship, and professional leadership. Furthermore, the increasing globalization and diversification of healthcare teams require perfusionists to demonstrate cultural competence, empathy, and emotional regulation in their daily practice [2].

Despite this, perfusion education programs have traditionally focused on clinical sciences, technical training, and simulation of procedural tasks. While these elements are foundational, the absence of structured soft skills training in curricula represents a critical gap. Current competency frameworks in allied health and nursing professions have already begun to prioritize non-technical skills as core learning objectives. It is timely and necessary for perfusion education to align with this direction [3, 4].

Incorporating soft skills into perfusion curricula can be achieved through various educational strategies. Simulation-based learning, particularly in team scenarios, provides a safe environment to practice communication and crisis management. Structured debriefing encourages reflective practice and feedback on team performance. Formal instruction on emotional intelligence, conflict resolution, and interprofessional collaboration can complement traditional didactic content. Additionally, mentorship programs and behavioral assessments during clinical rotations can reinforce these competencies.

There is also a strong case for integrating soft skills into certification and continuing professional development frameworks. Recognizing these competencies in accreditation standards would promote consistency across training institutions and support long-term professional growth [2–4].

In summary, while soft skills have always been critical to effective perfusion practice, they have often been developed indirectly through years of clinical experience. Given the increasing complexity of patient care, the multidisciplinary nature of surgical teams, and the expanding scope of perfusionist responsibilities, it is both appropriate and necessary to move toward a more intentional, structured approach to soft skills education. Preparing future perfusionists to excel not only in technical domains but also as communicators, collaborators, and adaptive professionals is essential to advancing both patient care and the profession itself.
